# How Healthy Are We? A National Study of Well-Being at Midlife

**Published:** 2004-06-15

**Affiliations:** Choice Care Occupational Medicine Atlanta, Ga

Every human being is the author of his own health or disease.

— Siddhartha Gautama, The Buddha, 563–483 BC

Many Americans might agree with this statement, but if asked to rate their own physical, mental, and health beliefs, what would their responses be? How would they respond at midlife? *How Healthy Are We? A National Study of Well-Being at Midlife answers these questions.*

In 1990, The John D. and Catherine T. MacArthur Foundation established the Research Network on Successful Midlife Development (MIDMAC), directed by one of the book’s editors, Orville Brim. The multidisciplinary network was tasked with examining adults aged 40 to 60 as part of the foundation’s series of studies on midlife development. MIDMAC identified the following goals:

- Establish specific indicators for a successful midlife.
- Establish a factual basis for the course of midlife.
- Identify factors that may influence the course of midlife development.
- Identify strategies that individuals may use to deal with midlife challenges.

MIDMAC developed a national survey known as MIDUS (Midlife Development in the United States) (available at http://midmac.med.harvard.edu/research.html) to help meet these goals. In *How Healthy Are We?*, Brim and colleagues present a collection of articles that summarize MIDUS survey findings.

The authors organize the text into four sections. Chapter 1 provides background on MIDMAC and the development of the MIDUS survey, including information on samples, design, measures, and content. Chapters 2 through 6 discuss assessments of physical health and the impact of age, gender, and socioeconomic status. Chapters 7 through 14 explore assessments of psychological well-being and quality of life. Chapters 15 through 21 focus on contexts that affect midlife experiences, including work, family, and geography. Each chapter provides background research, information on how MIDUS was designed and implemented, results, and a comparison between MIDUS and prior studies.

The audience for this text includes a broad range of scientists, such as sociologists, epidemiologists, mental health practitioners, psychologists, and anthropologists, reflecting the range of disciplines involved in developing the survey. In addition, the audience includes physicians, public health practitioners, and policy makers.

Of particular interest from a policy perspective is Chapter 17, which centers on work, family, and social class. The authors find that the lower the income level, the more likely it is that work is juxtaposed with poor social support, a chronically ill child, or other caretaking responsibility. Low-income jobs do not provide flexibility in sick leave or work hours, resulting in a disproportionate number of children who suffer from unmet health and developmental needs — and ultimately impacting our nation’s health.

Chapter 6, a discussion of menopause and the aging process, is another well-written and accessible chapter. The author finds that, for most women, menopause is a benign event unassociated with the severe physical and psychological distress traditionally portrayed.

Other interesting findings in the book include the following:

- Women report worse health than men, but they devote more effort to health maintenance and have greater longevity.
- The social/income gradient to ill health is greater in men than women and is independent of education.
- Adults rate the development of relationships with others as the most important factor in having a good life, followed by health, then family.
- While daily stresses or demands are greater in the lives of young and midlife adults compared to individuals in other life stages, midlife adults feel they have a greater level of mastery and control over stresses and demands than young adults do.

Overall, *How Healthy Are We?* answers the title question through research establishing midlife as a time when most adults have a positive view of physical health, have established supportive family relationships, are pleased with their financial situation, have a good quality of life, and have control and mastery of their work. Education, socioeconomic standing, race, and gender affect all of the above.

*How Healthy Are We?* also can serve as a basis for brainstorming community-based approaches for tackling chronic health issues and for devising new methods to encourage behavioral change. For example, an awareness of gender differences in health-maintenance behavior, noted in Chapter 1, could change approaches to promoting weight loss or smoking cessation for men and women.

Because the MIDUS survey included individuals aged 25–74, a more complete picture emerges on midlife in relation to the other life stages. *How Healthy Are We?* differs from other texts on midlife by presenting this expanded perspective. Some texts define midlife from a traditional psychological or biological perspective using staging or physical changes. Others take a health-management approach, focusing on specific aspects of midlife such as parenting or sexuality, or examining midlife in the context of larger societal trends such as divorce, two-income families, and longer life expectancy.

MIDMAC and MIDUS serve as excellent blueprints for an integrative approach to investigation. The book describes carefully developed pilot studies and creatively used satellite studies that provide information on a national level as well as on specific target groups. The public health profession could apply this approach to a wide range of problems.

The authors provide great insight into the physical, psychological, and social development that takes place during this important stage of life and establish a clear baseline for further exploration of midlife issues.

